# The 10th yearly volume of JPOR is complete

**DOI:** 10.17505/jpor.2024.27101

**Published:** 2024-12-13

**Authors:** Lars-Gunnar Lundh, Lars R. Bergman

**Affiliations:** 1 Department of Psychology, Stockholm University, Stockholm, Sweden; 2 Department of Psychology, Lund University, Lund, Sweden

The first issue of Journal for Person-Oriented Research (JPOR) was published in 2015, representing the first international journal specifically focused on person-oriented research. As defined under the JPORs Aim and Scope, person-oriented research is defined as

*theoretical, methodological, and empirical research that is guided by a research paradigm in which the individual is at focus and seen as a functioning totality.*

*This paradigm implies that theories and findings should be interpretable at the individual level and that patterns of individuals’ characteristics are of key interest.*

With this issue, the JPOR completes its tenth yearly volume. These ten volumes have included theoretical and methodological papers, as well as empirical papers using a wide variety of person-oriented approaches, spanning from cluster analysis, latent profile analysis and configurational frequency analysis, to intensive longitudinal studies using ecological momentary assessment (EMA) and time-series analyses to study development and change at the level of the individual.

The number of submissions to JPOR has been steadily increasing, although from low levels. During 2023, twenty manuscripts were submitted to the JPOR, and six of these were published, representing an acceptance rate of 30% and a rejection rate of 70%. During 2024, in total 29 manuscripts have been submitted when this is written (on December 10^th^ 2024).

## International Journal

An indication of JPOR’s status as an international scientific journal is its inclusion in the Scopus database, DOAJ and CrossRef, and in the full-text archive of PubMed. PubMed is a free full-text archive of biomedical and life sciences journal literature that is organized by the U.S. National Institutes of Health's National Library of Medicine (NIH/NLM), and the JPOR is included there since 2017. This means that all articles published in the JPOR from 2017 and onwards are freely available in full-text format at the PubMed site (https://pubmed.ncbi.nlm.nih.gov/).

The status of JPOR as a truly international journal is reflected in the number of countries represented among the authors who have contributed articles to the journal. As seen in Table 1, although most published articles are authored by researchers from the US, Sweden and the Netherlands, research from eleven other countries is also represented in the journal.

**Table 1:** Articles in JPOR originating from different countries

Country of origin^1^	Number of published articles
United states	37
Sweden	28
The Netherlands	11
Finland	4
Hungary	4
Germany	4
Austria	3
Israel	3
Denmark	2
Italy	2
Norway	1
Portugal	1
South Africa	1
Switzerland	1
**Total number of articles**	**102**

1 Country of the corresponding author.

## Increasingly Cited

Since 2019, the papers in JPOR are indexed by Scopus – which, however, also means that no data on papers published in the JPOR from 2015 to 2018 are available there. Nevertheless, Scopus data show that the impact of the JPOR is on the rise. Scopus reports a *CiteScore* for the JPOR of 2,9 during the years 2020-2023, which ranks JPOR as 690 among 1632 peer-reviewed psychological journals indexed by Scopus. This represents an increase from the previously reported 4-year period (2019-2022). The *CiteScore* is calculated by dividing the number of citations during a four-year period by the number of all papers published during this period. Thus, JPOR’s *CiteScore* of 2,9 is based on 80 citations of the totally 28 papers published in JPOR during 2020-2023.

An important limitation of measures such as *CiteScore* (as computed by Scopus) and *Impact* (as computed by the Web of Science), however, is that they only measure the mean number of citations that the articles in a journal receive *during the next few years after publication*. This means that these kind of measures have a bias for research that is in line with what is scientifically “popular” at the time (and that can therefore be expected to get more citations in the short run), whereas they have a bias against research that is slower to gain momentum because it presents new ideas and new findings that go against the current tide (and are therefore less likely to received citations in the short run).

**Figure 1:**
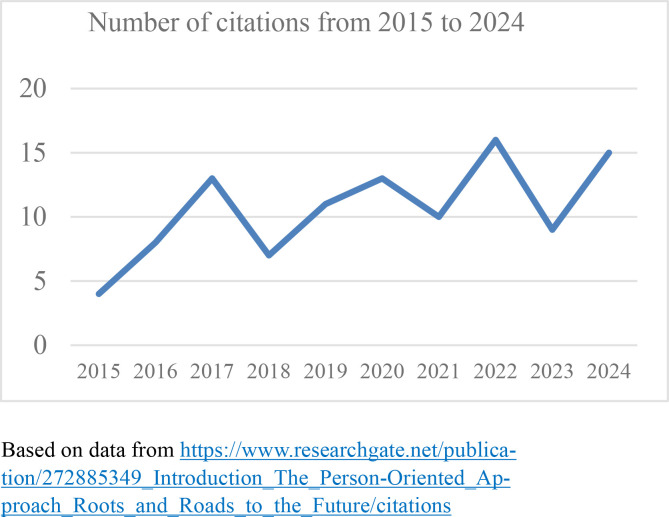
Number of citations 2015-2024 of “The person-oriented approach: Roots and roads to the future” (Bergman & Lundh, 2015), as reported by Research Gate

Some papers continue to be cited year after year, and even show rising trends in the number of citations per year. To take an example from the JPOR, the introductory article from its very first issue, *The person-oriented approach: Roots and roads to the future* (Bergman & Lundh, 2015), has so far been cited 106 times, as reported by ResearchGate^1^ on December 10^th^ 2024. Moreover, as seen in Figure 1, the number of citations has not slowed down, but has rather tended to increase during the 10-year period since publication. Over the first 5-year period, the number of citations was 43, which means an average of 8,6 citations per year. During the recent 5-year period, the number of citations was 63, which means an average of 12,6 citations per year.

**Figure 2:**
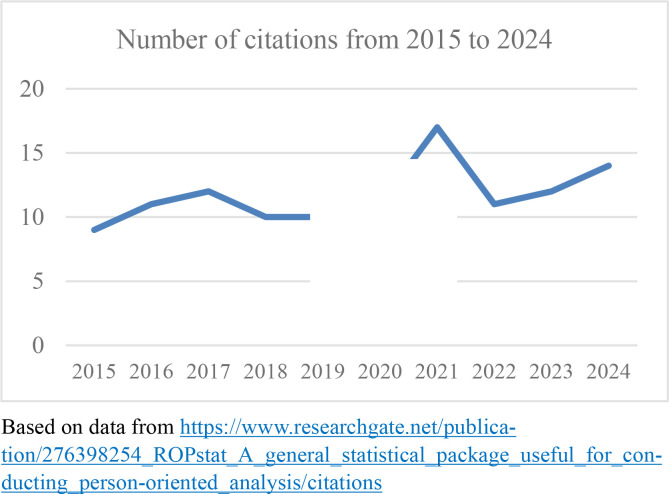
Number of citations 2015-2024 of “ROPstat: A general statistical package useful for conducting person-oriented analyses” (Vargha et al., 2015), as reported by Research Gate.

Of the other articles that were published in the very first issue of the JPOR, one has had even more citations: Vargha et al.’s (2015) *ROPstat: A general statistical package useful for conducting person-oriented analyses*, which had been cited 117 times by December 10^th^ 2024, as reported by ResearchGate. In their paper, Vargha et al. (2015) described the general features and the main structure of a new user-friendly statistical package for person-oriented analyses, ROPstat, which has shown to be highly helpful for researchers who want to carry out more advanced forms of person-oriented analysis (e.g., cluster analysis) than those available in other statistical packages. As seen in Figure 2, the number of citations here has also tended to increase during the 10-year period since publication. Over the first 5-year period, the number of citations was 52, which means an average of 10,4 citations per year. During the recent 5-year period, the number of citations was 65, which means an average of 13 citations per year.

**Figure 3:**
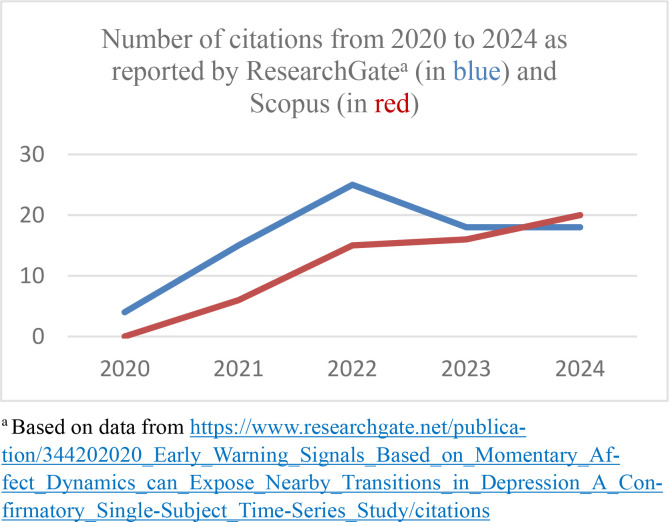
Number of citations 2020-2024 of “Early warning signals based on momentary affect dynamics can expose nearby transitions in depression: A confirmatory single-subject time-series study” (Wichers et al., 2020).

As to number of citations of JPOR articles published during the last 5-year period, the most cited article is Wichers et al.’s (2020) *Early warning signals based on momentary affect dynamics can expose nearby transitions in depression: A confirmatory single-subject time-series study*. Their results showed the potential of early warning signals to improve personalized risk assessment in the field of psychiatry. Because this paper was published in 2000, and is therefore included in the Scopus database, it is possible to compare the number of citations reported by ResearchGate with those reported by Scopus. As seen in Figure 3, ResearchGate reported 80 citations of this paper on December 10^th^ 2024, whereas Scopus reported 57 citations. The data from Scopus here shows a continuously rising trend in citations per year.

## Called for: Papers on the Application of Non-Linear Dynamic System Modelling

As can be seen from this short overview of the first ten years of the JPOR, we believe that our journal has been successful in providing the scientific community with an outlet for modern person-oriented research. This is much needed considering that the journal is the only one focusing on publishing in this increasingly important research field. The journal has covered a large number of different directions within the broad spectrum of person-oriented approaches, both methodological, theoretical, and empirical. We appear to be on good track, and our hopes are high for the future of the journal.

However, we should also ask what is missing and what should be encouraged for future publications. One important such area concerns the dearth of articles dealing with the application of nonlinear dynamic system modelling. Certainly, some articles in this area have been published in our journal but only one article deals with the construction and use of a nonlinear dynamic model (NOLIDS) that can function as a template for other researchers modelling their data using this approach (Grip & Bergman, 2016). This is insufficient, considering the great potential of NOLIDS. This type of model is especially well aligned to the theoretical conceptualizations and assumptions accepted by most developmen-tal researchers. These tenets are strongly violated when using standard statistical analyses, for instance structural modelling, for analyzing developmental data when the purpose is to understand individual development. Of course, one reason for this state of affairs is that NOLIDS methodology is technically quite difficult to learn and apply and is not within the field of expertise of the ordinary methodologist or statistician. But NOLIDS has proved to be a very successful approach in the natural sciences, so collaboration with experts in this methodology should be considered.

## The Present Issue

The present issue of JPOR is rather representative both of the kind of papers that are typically published in our journal, and of the international breadth of the contributions. In the first paper (Olthof et al., 2024), a group of researchers from the Netherlands focus on two common phenomena in research with Ecological Momentary Assessment (EMA) data – non-stationarity and outlying values – arguing that these may carry important information about the individual and should not be seen merely as nuisances to be dealt with. They make the interesting point that complementing EMA time series with contextual information and qualitative data can be essential to genuinely understand these phenomena.

The second contribution is a brief theoretical paper, where the American researcher James Lamiell (2024b) raises the question whether population-level research really belongs to psychological science. His suggestion is that it should rather be seen as a form of *demographic* research (psycho-demography). This represents a thought-provoking contribution to an ongoing debate on the nature of psychological science, initiated by Lundh (2023), who argued that population psychology *does* represent one branch of psychological science (together with person psychology and mechanism psychology as two other main branches). Additional contributions to this debate have been made by Hofman et al., (2024), Lamiell (2024a), Lundh (2024), and Nilsson (2024).

The third paper, written by an Italian group of researchers (Sica et al., 2024), uses latent profile analysis to analyze patterns of meaning in life, optimism, future orientation, and wellbeing in a study of coping during the Covid-19 pandemic. Interestingly, their paper also contains a methodological comparison between their latent profile analysis and a cluster analysis on the same data.

## Open access

This article is distributed under the terms of the Creative Commons Attribution 4.0 International License (http://creativecommons.org/licenses/by/4.0/), which permits unrestricted use, distribution, and reproduction in any medium, provided you give appropriate credit to the original author(s) and the source, provide a link to the Creative Commons license, and indicate if changes were made.

## Footnotes

1 *ResearchGate* is a European social networking site for scientists and researchers which traces citations of all articles that are shared there. One advantage of *ResearchGate*, as compared with *Scopus*, is that it reports citations from the start of JPOR in 2015, whereas JPOR was not included in the *Scopus* database until 2019.
